# Development of a compound hazard risk index for natural and nuclear disasters and establishment of a prefecture-wide support system in Aomori

**DOI:** 10.3389/fpubh.2025.1750224

**Published:** 2026-01-20

**Authors:** Ryusei Maekawa, Takakiyo Tsujiguchi, Masato Naraoka, Kanako Yamanouchi, Katsuhiro Ito

**Affiliations:** 1 Graduate School of Health Sciences, Hirosaki University, Hirosaki, Japan; 2 Hirosaki University Radiation Emergency Medicine and Cooperation Promotion Education Center for Disaster and Radiation Emergency Medicine, Hirosaki, Japan; 3 Advance Emergency and Critical Care Center, Hirosaki University Hospital, Hirosaki, Japan

**Keywords:** damaged hospital continuation support, disaster medicine, hospital function maintenance, nuclear disaster, pre-disaster list

## Abstract

**Background:**

Japan is highly susceptible to various disasters, as exemplified by the recent Noto Peninsula Earthquake. This study targeted Aomori Prefecture, located at the northernmost tip of Japan’s main island, which faces the compound risks of natural and nuclear disasters.

**Methods:**

A vulnerability assessment was conducted for 108 medical institutions using a seven-item risk index to create a “pre-disaster list” (PL). Simulations based on Cabinet Office damage projections were integrated to develop a Hospital Support List (HSL).

**Results:**

The simulations revealed a potential need for the emergency evacuation of up to 2,859 patients. Critical vulnerabilities were identified across multiple indicators, including structural integrity, lifelines (power, oxygen, and water supply), and radiation protection systems.

**Conclusion:**

The findings indicate an urgent need to clarify the cooperative roles of Disaster Medical Assistance Teams (DMAT) and the Nuclear Emergency Medical Assistance Team (NEMAT). Establishing a comprehensive support system is essential for maintaining hospital functions and mitigating damage in future compound disasters.

## 1 Introduction

Owing to its natural conditions, Japan is susceptible to various disasters and has experienced damage, including the recent Noto Peninsula Earthquake (1, 2). It is not uncommon for nuclear power plants to be located in areas with high risks of earthquakes and tsunamis, necessitating the consideration of countermeasures for concurrent nuclear disasters during peacetime. If natural and nuclear disasters occur simultaneously, the regional medical support system may be severely impacted.

The framework for disaster management in Japan is established upon the Basic Act on Disaster Management and the Act on Special Measures Concerning Nuclear Emergency Preparedness. Within this legal framework, the Nuclear Emergency Preparedness Guidelines underwent a revision in 2015 in response to the Fukushima Daiichi Nuclear Power Station accident, which necessitated the enhancement of both evacuation planning and medical support structures. In accordance with these developments, recent disaster medical response strategies have prioritized the maintenance of hospital functions from the acute phase of a disaster (3).

Accordingly, there has been an increase in cases in which Disaster Medical Assistance Teams (DMAT) and other response teams provide lifeline support, such as fuel for private power generators and water supplies, as well as support for the emergency evacuation of patients owing to lifeline disruptions (4–6).

However, accurately determining “which medical institution needs what supplies,” “support priorities,” and “which facilities to evacuate” is difficult with limited information and time. When complex events involving nuclear disasters are intertwined, delays in support are a concern. To address this, the Damaged Hospital Continuation Support (DHCoS) simulation has been implemented to enable precise and rapid support for maintaining hospital functions during disasters. The DHCoS is an initiative that involves collecting data on the seismic resistance and lifeline functions of hospitals during peacetime to create a “Pre-disaster List” (PL) that organizes the vulnerabilities of each hospital. Based on this PL and disaster damage scenarios, the allocation of DMATs and prioritization of medical support are simulated in advance (7).

Although the development of PLs to understand the lifeline status of medical institutions is being promoted in each prefecture, there is currently significant regional variation in their adoption, and they are insufficiently established. Furthermore, research on medical support during compound disasters, including nuclear incidents, is limited. While conventional disaster medicine research has primarily focused on natural disasters, considerations specific to nuclear disasters, such as difficulties in evacuating and securing support personnel, have been insufficient (7).

Therefore, the aim of this study was to facilitate prompt and smooth support during disasters by assuming the occurrence of both natural and nuclear disasters. To this end, we first analyzed the disaster risks of medical institutions within the prefecture in detail to create a PL that contributes to hospital function maintenance support. Furthermore, by simulating disaster risks, we examined effective support measures and identify challenges, with the goal of constructing a support system model that is applicable to other regions with nuclear-related facilities.

## 2 Materials and methods

### 2.1 Data collection from medical institutions in Aomori prefecture

This study was conducted in 108 medical institutions in Aomori Prefecture, located at the northernmost tip of Japan’s main island and home to nuclear facilities. The institutions included 12 disaster base hospitals, 2 DMAT-designated medical institutions, and other general hospitals. Using the Emergency Medical Information System (EMIS), we collected the information presented in Table 1. This information encompasses facility basics (number of beds, presence and capacity of water receiving and elevated tanks, well facilities, and average daily water usage), seismic diagnostic results (presence of seismic resistance and identification of non-resistant facilities), inventory of medical equipment dependent on lifelines (ventilators, dialysis machines, incubators, etc.), private power generators (presence, fuel volume, operating time, and fuel type), facility damage assumptions (flood and tsunami inundation depths, and classification of landslide risk), and radiation protection measures (building shielding [wall thickness] and heating, ventilation, and air conditioning [HVAC] systems for contamination control). These data were used to assess the disaster vulnerability of the medical institutions and to inform the development of pre-disaster plans and support measures for maintaining hospital functions (Table 1). Detailed information regarding each medical institution was supplemented with the cooperation of the Aomori Prefectural Government’s Health and Medical Affairs Division and DMAT members active in the prefecture to improve data accuracy.

**Table 1:** Information items were collected from the target medical institutions.

Information category	Details of collected items (unit)
Facility basic information	Number of beds
	Presence/capacity of water receiving tank (t)
	Presence/capacity of elevated water tank (t)
	Presence of well facilities
	Average daily water usage on weekdays/holidays (t)
Seismic diagnosis	Presence of seismic resistance
	Identification of non-seismically resistant facilities
Inventory of lifeline-dependent equipment	Number of ventilators
	Number of dialysis machines
	Number of incubators
	Number of other equipment
Private power generators	Presence of private generator
	Fuel volume (kL)
	Operating hours (h)
	Fuel type
Facility damage assumption	Assumed flood inundation depth (m)
	Assumed tsunami inundation depth (m)
	Type of landslide risk
Radiation protection measures	Building shielding (wall thickness)
	HVAC systems

### 2.2 Risk assessment and creation of the PL

The PL in this study was created based on information collected from medical institutions, using seven risk indicators. The specific risk indicators are (i) risk of building collapse, (ii) risk of flooding, (iii) possibility of power loss, (iv) instability of electricity supply, (v) instability of oxygen supply, (vi) instability of water supply, and (vii) radiation protection system.

The risk of building collapse was considered high for facilities with no or unconfirmed seismic resistance. The risk of flooding (including landslides, and avalanches) was deemed high for facilities located in areas with an expected inundation depth of 3 meters or more, or within a landslide (avalanche) warning area. The possibility of power loss was high for facilities without a private generator or those at risk of power loss due to flooding. Instability of electricity supply was judged as high for facilities with less than a day’s worth of generator fuel and possessing ventilators. Instability of oxygen supply was high for facilities with ventilators, and instability of water supply was high for those with dialysis machines. Indicators (i)–(vi) were set with reference to guidelines from the DMAT Secretariat of the Ministry of Health, Labor, and Welfare, while indicator (vii) was established as an original evaluation item based on the nuclear disaster risk within Aomori Prefecture (Table 2).

**Table 2:** Risk assessment details for each risk indicator.

Risk assessment				Item			
	(i)	(ii)	(iii)	(iv)	(v)	(vi)	(vii)
High	〇	No or uninspected seismic resistance	Assumed inundation ≥3 m or within a landslide (avalanche) warning area	No private generator or possibility of power loss due to flooding	Generator fuel for ≤1 day and has ventilators	Has ventilators	Has dialysis machines
Low	―	Does not meet the above criteria	Does not meet the above criteria	Does not meet the above criteria	Does not meet the above criteria	Does not meet the above criteria	Does not meet the above criteria

To facilitate prompt protective measures during a nuclear disaster, “Nuclear Emergency Preparedness Zones” are established around nuclear facilities. Specifically, for nuclear power reactor facilities, a Precautionary Action Zone (PAZ), where immediate evacuation is assumed, is set within an approximate 5-km radius of the facility. An Urgent Protective Action Planning Zone (UPZ), in which evacuation and shelter are prepared for depending on the situation, is set within an approximate 30-km radius (8, 9). Medical institutions within the PAZ and UPZ are required to implement radiation protection measures. However, some facilities have inadequate measures. Therefore, by referencing the risk assessment indicators for radiation protection proposed by Tsujiguchi et al. (7), we evaluated the presence or absence of radiation protection measures (building shielding and HVAC systems) at each medical institution. Consequently, medical institutions located within the PAZ or UPZ that have not implemented radiation protection measures were defined as the “high-risk group.” Based on these seven indicators, including radiation protection risk, the disaster risk of each medical institution was assessed (Table 2).

### 2.3 Implementation of the DHCoS (simulation of hospital function maintenance support based on a disaster scenario)

**Figure 1:**
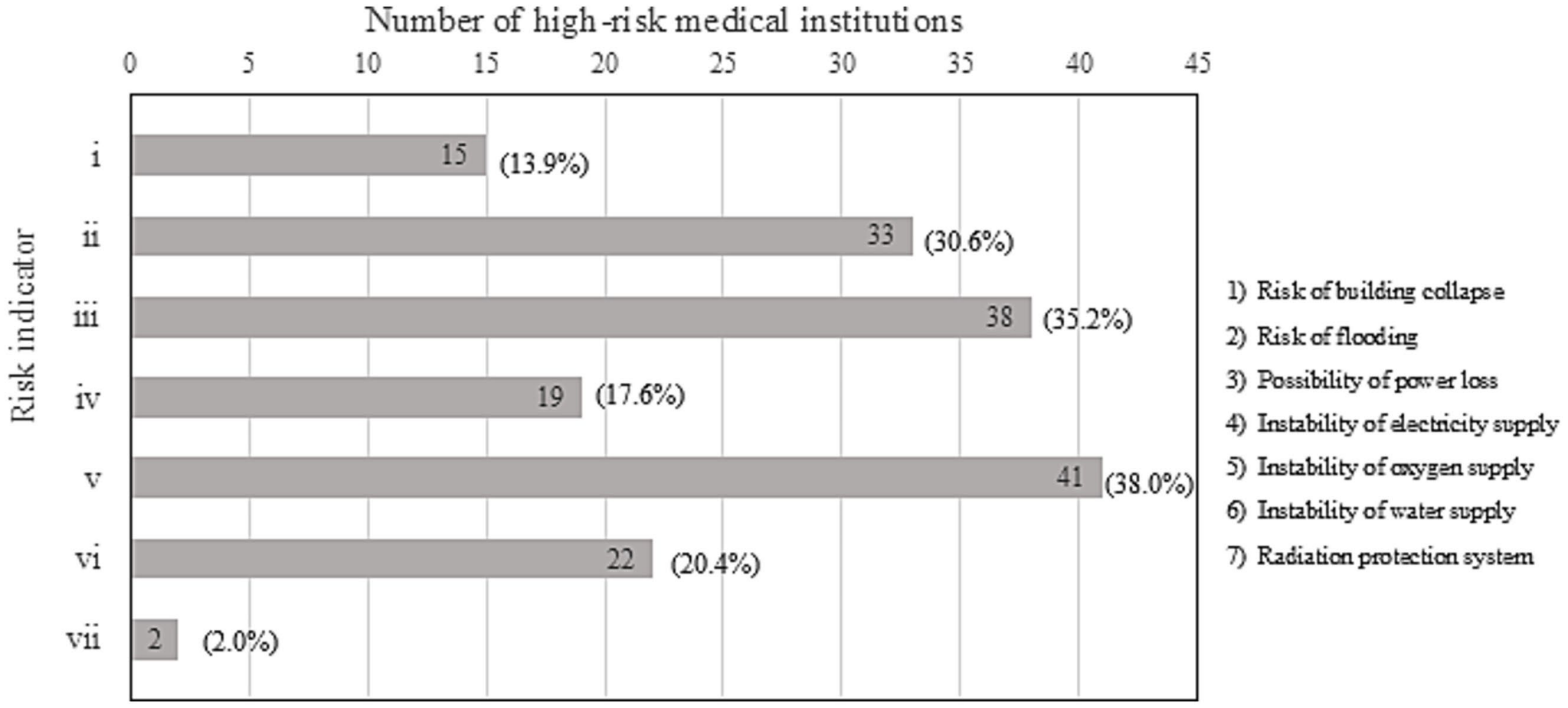
Conceptual diagram of DHCoS.

In this study, a simulation of hospital function maintenance support was conducted based on a disaster scenario (DHCoS). A conceptual diagram of the DHCoS used in this study is shown in Figure 1. The DHCoS utilized a Hospital Support List (HSL), which combines the PL with a specific damage scenario.

#### 2.3.1 Creation of the HSL

The HSL was created to implement the DHCoS. The HSL is a list of vulnerable hospitals under conditions of collapse at a seismic intensity of 6-lower or greater, flooding from tsunamis, power outages, and water supply disruptions, reflecting post-disaster damage conditions onto the PL. Because actual damage data are not available, we conducted a simulation by incorporating the “Damage Estimation for a Great Earthquake along the Japan Trench and Kuril-Kamchatka Trench” (10) published by the Cabinet Office in 2021 and the scenario for the large-scale earthquake medical activity drill of fiscal year 2025. Here, even if a medical institution was judged as high-risk on the PL, it was excluded from the support target based on the simulated damage situation to identify facilities with a high probability of needing support. The exclusion criteria are listed in Table 3.

**Table 3:** Exclusion criteria for the pre-disaster list.

Risk item	Exclusion criteria
Collapse	High-risk collapse facilities located in areas where the seismic intensity is expected to be 5-upper or less
Flooding	High-risk flooding facilities located in areas where the inundation depth is expected to be less than 3 m
Power loss	High-risk power loss facilities located in areas where no power outage or flooding is expected

#### 2.3.2 Assumption of disaster response based on the HSL

Based on the created HSL, we conducted a study assuming actual disaster response, such as determining support priorities in the acute phase of a disaster and identifying medical institutions that require evacuation support.

## 3 Results

### 3.1 Creation of the PL based on collected information from medical institutions

The PL of medical institutions created is shown in Supplementary Table 1.

### 3.2 Creation of the HSL and risk analysis of medical institutions

The HSL was created by cross-referencing the created PL with the disaster scenario (Supplementary Table 2). As a result, of the included 108 medical institutions, the number of facilities judged as high-risk for each risk indicator was as follows: 15 institutions for building collapse risk (13.9%), 33 for flooding risk (30.6%), 38 for power loss risk (35.2%), 19 for unstable electricity supply risk (17.6%), 41 for unstable oxygen supply risk (38.0%), 22 for unstable water supply risk (20.4%), and 2 for radiation protection risk (1.9%).

A comparison between the PL and the HSL, which incorporates disaster damage projections, reveals that while the risks of “collapse” and “unstable water supply” decreased following the application of exclusion criteria, the number of institutions at high risk for “flooding” and “power loss” increased significantly from 19 to 33 and from 28 to 38, respectively. This trend is attributable to the adoption of a large-scale earthquake scenario, which reflects extensive tsunami damage exceeding the projections of conventional hazard maps. Furthermore, the HSL accounts for practical functional failures during a disaster—such as generator malfunction due to inundation or fuel depletion—that cannot be captured solely by the presence of equipment as evaluated in the PL. These findings demonstrate that overlaying specific disaster scenarios effectively manifests the latent vulnerabilities of medical institutions, thereby identifying those in urgent need of prioritized support.

**Figure 2:**
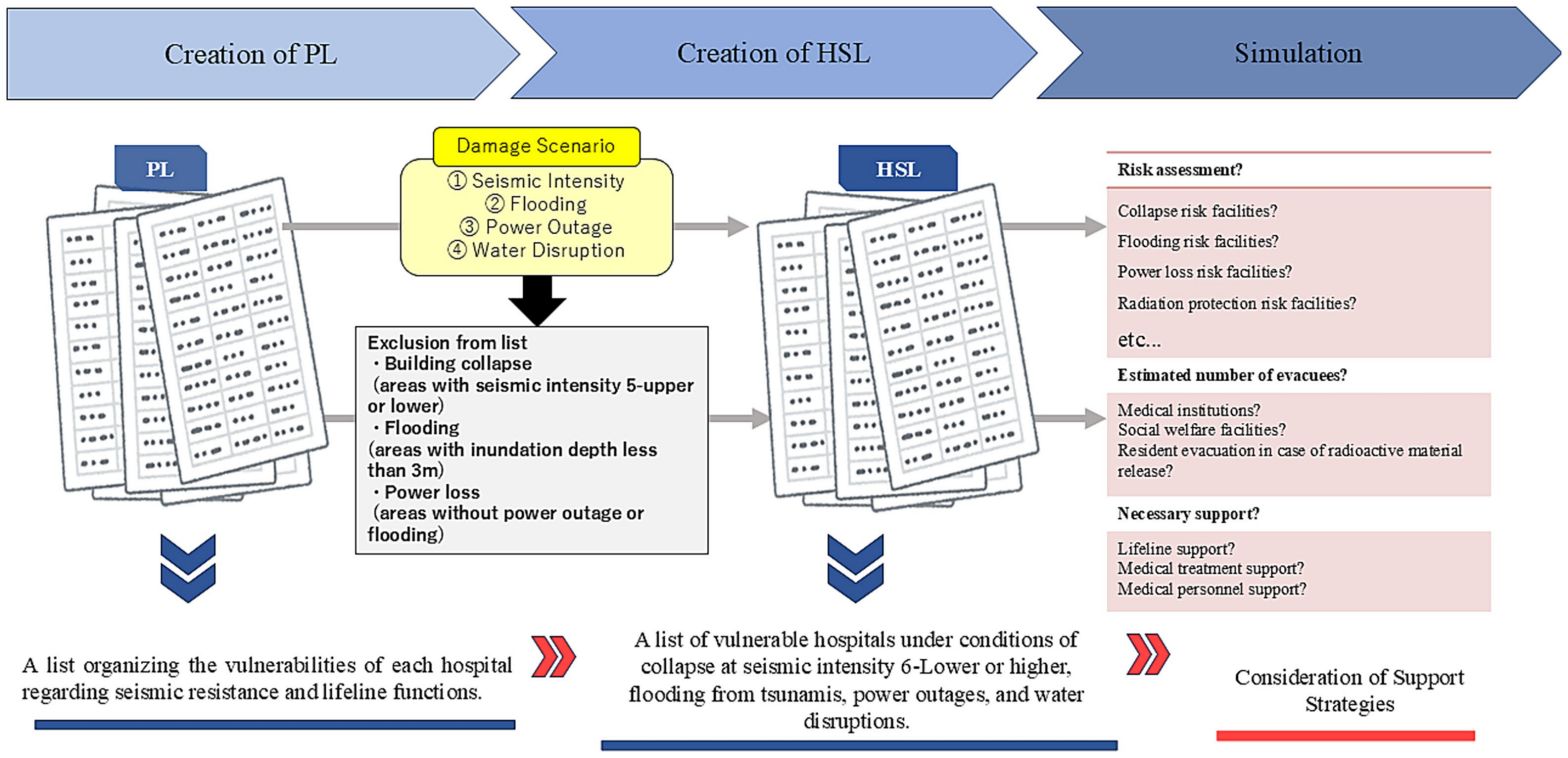
Number of high-risk medical institutions for each risk indicator (108 facilities in Aomori prefecture).

Figure 2 is a bar graph showing the number of medical institutions judged as high-risk for each risk indicator. It indicates that risks related to oxygen supply instability and power loss are relatively common.

### 3.3 Estimation of support needs in a disaster

“Collapse” and “flooding” are critical hazards affecting the structural safety of medical institutions, and high-risk facilities require prompt evacuation actions (11). In this study, 36 institutions were identified as high-risk in either of these categories (with overlaps). If these institutions required evacuation while their beds were at full capacity, a maximum of 2,859 patients would require emergency evacuation. Of these, 22 institutions (61.1%) are concentrated in the Hachinohe–Kamikita medical area, with a maximum of 2,188 evacuees.

Furthermore, two medical institutions were identified as having inadequate radiation protection measures (based on an original item created in this study), one of which is included in the aforementioned 36 institutions. The Nuclear Emergency Response Guidelines establish emergency action levels (EAL) corresponding to the anticipated stages of radioactive material release, divided into three stages: “Alert (AL),” triggered by events such as a seismic intensity of 6-lower or greater; “Site Area Emergency (SE),” for events like a station blackout; and “General Emergency (GE),” when the actual release of radioactive materials becomes imminent. Evacuation preparations begin at the AL stage. At the SE stage, actual evacuation and preparations for stable iodine distribution begin. At the GE stage, prompt evacuation or sheltering is required for residents within the PAZ and UPZ (12, 13). The two high-risk medical institutions identified as having inadequate radiation protection measures had a total of 554 beds and were highly likely to be classified as “requiring evacuation” based on the EAL even at the SE stage, making a maximum of 554 patients potential evacuation targets. Moreover, one of the two institutions is also a target for emergency evacuation; thus, it has the highest priority for evacuation support among all medical institutions in the prefecture.

Excluding the 36 medical institutions subject to emergency evacuation, the remaining 72 facilities were analyzed for overlapping high-risk items to serve as a reference for prioritizing support and allocating resources during a disaster. Supplementary Table 2 summarizes the degree of overlap of high-risk items for each facility.

As shown in Table 4, 58 institutions (80.6%) had zero or one high-risk item, whereas 14 institutions (19.4%) had two or more high-risk items.

**Table 4:** Overlap of high-risk items with risk indicators.

Number of high-risk items	Number of medical institutions
0	30
1	28
2	10
3	3
4 or more	1

**Figure 3:**
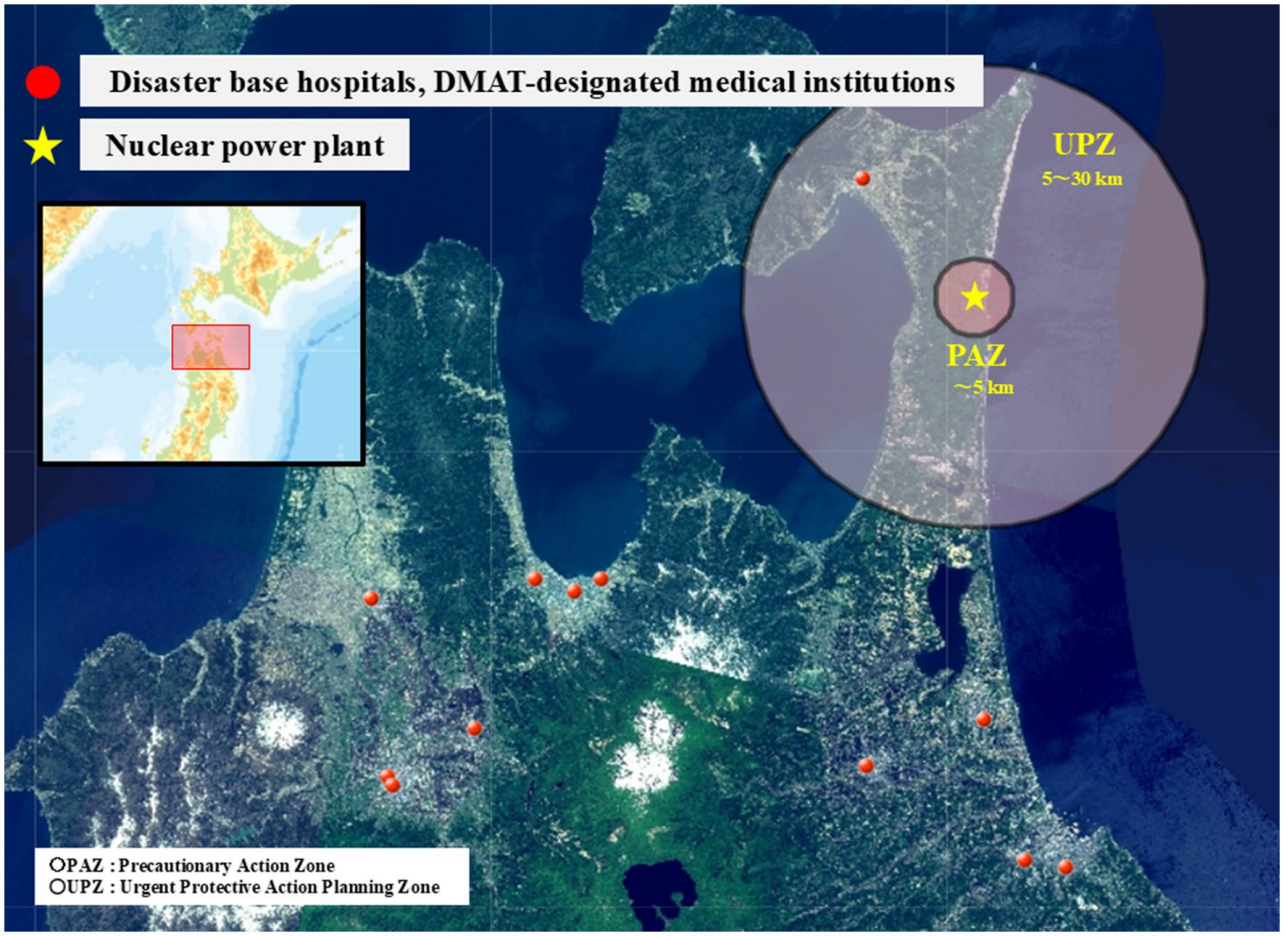
Geospatial distribution of disaster base hospitals and DMAT-designated medical institutions in Aomori Prefecture relative to nuclear emergency preparedness zones.

**Figure 4:**
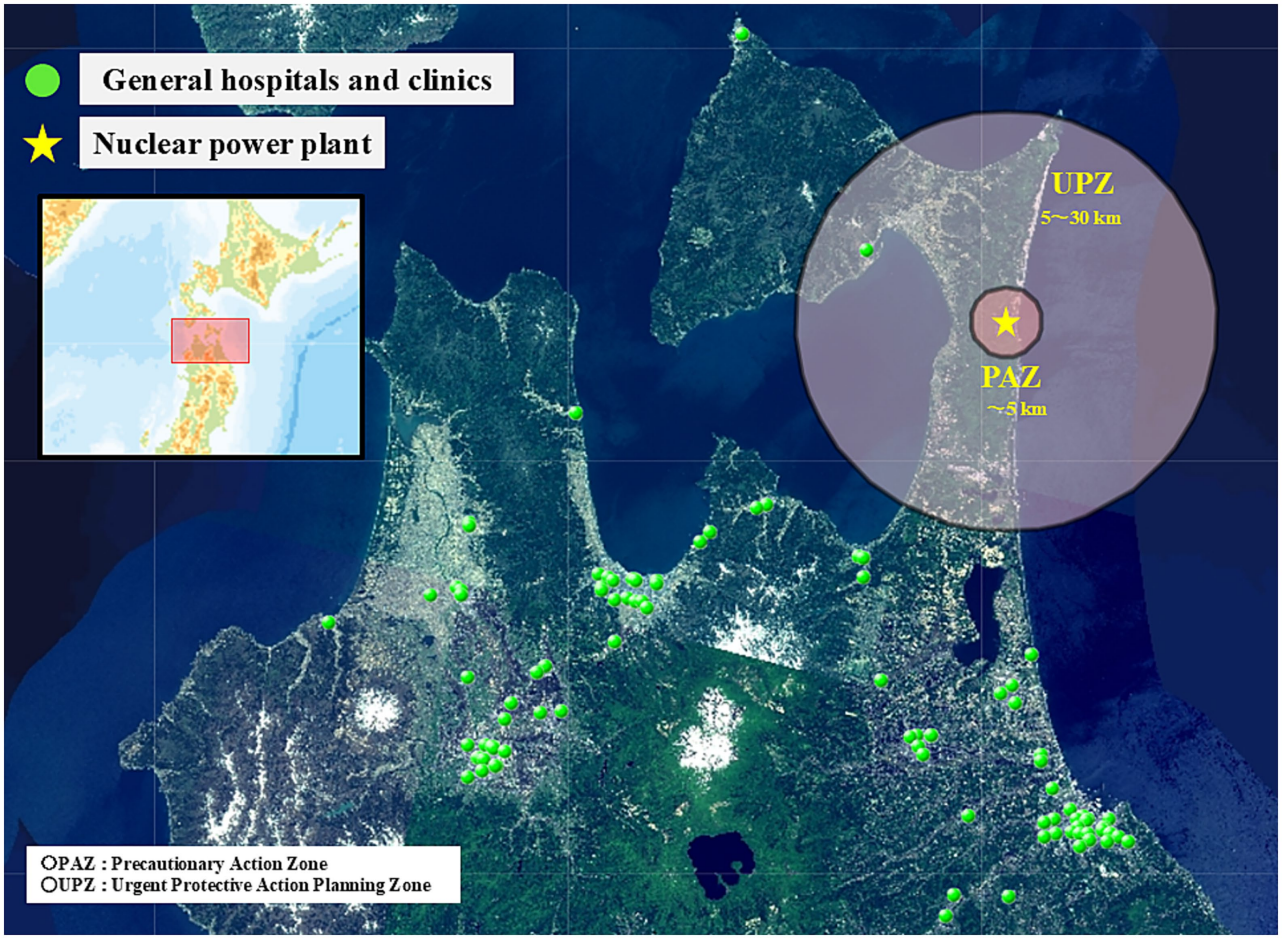
Geospatial distribution of general hospitals and clinics in Aomori prefecture relative to nuclear emergency preparedness zones.

Furthermore, the geospatial distributions of these medical institutions in Aomori Prefecture are shown in Figures 3, 4. Figure 3 illustrates the distribution of disaster base hospitals and DMAT-designated medical institutions relative to the Nuclear Emergency Preparedness Zones. Figure 4 depicts the distribution of general hospitals and clinics relative to these same zones.

## 4 Discussion

In this study, we created a unique PL based on seven risk indicators, assuming a compound disaster in Aomori Prefecture, to enable prompt and smooth support during such events. The creation of the PL enabled the quantitative assessment of each medical institution’s vulnerability. Furthermore, a disaster scenario simulation using this list revealed multiple vulnerabilities faced by medical institutions across the prefecture.

First, regarding structural vulnerability, 36 institutions were identified as being at high risk of “collapse” or “flooding,” with an estimated maximum of 2,859 requiring emergency evacuation. A significant number of these patients were concentrated in 22 institutions in the Hachinohe–Kamikita region. The importance of hospital evacuation has been widely recognized since the Great East Japan Earthquake, and patient transport by DMATs has been conducted during actual disasters (14). However, reports indicate that patient transport is difficult in large-scale disasters (15), with cases of significant disruptions to land transportation owing to road network disturbances (16–18). Therefore, targeted medical institutions must plan to secure air transport options in addition to land transport means such as ambulances, contracted buses, and taxis during peacetime.

Regarding lifeline vulnerabilities, 41 institutions were identified as being at risk of unstable oxygen supply, and 22 institutions were at risk of unstable water supply. This risk information, which is directly linked to life support, serves as an extremely effective basis for DMATs and other response teams to determine support priorities and specifically identify “where” and “what” to provide during the acute phase of a disaster. Additionally, as part of peacetime preparation, it is essential to develop a contact list covering all high-risk medical institutions and to establish a support-receiving system on the medical institution side. Although the importance of such a system has been pointed out, according to a study by Tanno et al. (19) the disaster support-receiving systems in the prefecture’s hospitals remains insufficient. There is an urgent need to establish a system that enables smooth coordination by identifying the contact person for support requests and specifying the required support details.

Furthermore, we assessed the risk of a compound disaster, in which natural and nuclear disasters occur simultaneously. The results revealed two medical institutions (totaling 554 beds) within the PAZ/UPZ with inadequate radiation protection systems. These institutions also had the aforementioned structural vulnerabilities. During the Fukushima Daiichi Nuclear Power Plant accident, some medical staff left because of anxiety about radiation, leading to severe staff shortages (20).

DMAT is defined as a team capable of providing medical care in disaster-affected areas during the acute phase of natural disasters; however, their operational guidelines generally assume activities in areas where safety is ensured (21, 22). Consequently, their standard equipment does not account for sufficient radiation protection for activities under high-dose conditions, making operations within PAZ/UPZ areas with radiation contamination risks extremely difficult. Thus, NEMAT’s support is indispensable in compound disasters. On the other hand, while NEMAT consists of specialists in radiation medicine, its primary mission is to support nuclear disaster base hospitals, and the number of available teams is limited (23). As a result, their absolute numbers are significantly insufficient to simultaneously handle activities requiring extensive logistics and manpower, such as the evacuation and transport of numerous inpatients or responding to building damage caused by earthquakes.

Furthermore, while a national nuclear disaster prevention system for evacuation exit screening is being developed, a medical support system for areas requiring sheltering or relocation remains underway (23). Therefore, considering the need to respond to both the surrounding residents and patients from these institutions, managing such situations under the current system would be extremely difficult. This situation gives rise to areas where existing frameworks are unable to provide sufficient assistance during a compound disaster, such as when a hospital is physically damaged by an earthquake while also facing radiation risks. It is therefore essential to transcend the traditional departmentalized boundaries between DMAT (natural disasters) and NEMAT (nuclear disasters) to reconstruct a comprehensive support system.

The PL and HSL developed in this study are highly effective in proactively identifying medical institutions at risk of facing a critical lack of support. Rather than being a mere inventory of equipment availability, these lists enable the visualization of when and how specific institutions will face critical functional failures by overlaying geographical conditions with disaster damage estimations.

Based on the findings from the PL and HSL, we should prioritize the reconstruction of a concrete support system aimed at mitigating and suppressing future disaster damage; specifically, this entails the preferential allocation of DMAT equipped with radiation protection gear and specialized expertise to identified high-risk medical institutions, alongside the establishment of an effective command system through cross-organizational mutual support agreements, such as frameworks where DMAT operate under the management and advice of NEMAT.

Overall, to improve disaster response capabilities in Aomori Prefecture, it is essential to conduct a multifaceted vulnerability assessment of medical institutions during peacetime and to formulate concrete advance plans based on these assessments. Future tasks should include the reconstruction of a comprehensive support system, including a clear division of roles between the DMAT and the Nuclear Emergency Medical Assistance Team, and the formulation of rapid coordination plans at the prefectural and municipal levels. Additionally, the PL created in this study should be updated regularly so that it can be continuously utilized for facility renovations and system strengthening in medical institutions. Furthermore, since maintaining the function of social welfare facilities is extremely important during a disaster, it is necessary to expand risk assessment in the future to include welfare facilities. However, a limitation of the present study is the omission of risk assessment for social welfare facilities, which must be addressed in future work to ensure a comprehensive disaster response. Future research should also focus on the clear division of roles between the DMAT and the Nuclear Emergency Medical Assistance Team and the formulation of rapid coordination plans at the prefectural and municipal levels.

## Data Availability

The original contributions presented in the study are included in the article/Supplementary material, further inquiries can be directed to the corresponding author.
