# Supplementary material for: Development of a compound hazard risk index for natural and nuclear disasters and establishment of a prefecture-wide support system in Aomori
Source: Front Public Health. 2026 Jan 20;13:1750224. doi: 10.3389/fpubh.2025.1750224 (PMC12864454; doi:10.3389/fpubh.2025.1750224)
Supplement: Supplementary file 1 [file Table_1.docx]

Supplementary Material

# Supplementary Tables

Supplementary Table 1. Pre-disaster list of medical institutions

| **Medical institutions** | **Risk indicator** | | | | | | | **Medical institutions** | **Risk indicator** | | | | | | |  |
| --- | --- | --- | --- | --- | --- | --- | --- | --- | --- | --- | --- | --- | --- | --- | --- | --- |
|  |  |  |  |  |  |  |  |  |  |  |  |  |  |  |  |  |
|  | **i** | **ii** | **iii** | **iv** | **v** | **vi** | **vii** |  | **i** | **ii** | **iii** | **iv** | **v** | **vi** | **vii** |  |
|  |  |  |  |  |  |  |  |  |  |  |  |  |  |  |  |  |
| 1 | 〇 | 〇 |  |  | 〇 | 〇 | 〇 | 55 |  |  |  |  |  |  |  |  |
| 2 |  |  |  |  |  |  |  | 56 |  |  |  |  |  |  |  |  |
| 3 | 〇 |  |  | 〇 | 〇 |  |  | 57 |  |  |  |  | 〇 | 〇 |  |  |
| 4 |  |  |  |  | 〇 | 〇 |  | 58 |  |  |  | 〇 | 〇 | 〇 |  |  |
| 5 | 〇 |  | 〇 |  |  |  |  | 59 |  |  |  |  |  |  |  |  |
| 6 |  |  |  |  |  |  |  | 60 |  |  |  |  |  |  |  |  |
| 7 | 〇 |  |  |  |  |  |  | 61 |  |  |  |  |  |  |  |  |
| 8 |  |  |  |  | 〇 | 〇 |  | 62 |  |  |  |  |  |  |  |  |
| 9 |  | 〇 | 〇 |  |  |  |  | 63 |  |  |  |  |  |  |  |  |
| 10 |  |  |  |  |  |  |  | 64 |  |  |  |  | 〇 | 〇 |  |  |
| 11 |  |  |  |  |  |  |  | 65 |  |  |  |  | 〇 | 〇 |  |  |
| 12 |  | 〇 | 〇 | 〇 | 〇 |  |  | 66 |  |  |  |  |  |  |  |  |
| 13 |  |  |  |  |  |  |  | 67 |  |  |  |  |  |  |  |  |
| 14 |  |  |  |  |  |  |  | 68 |  |  |  |  | 〇 |  |  |  |
| 15 |  |  |  |  | 〇 |  |  | 69 |  |  |  |  |  |  |  |  |
| 16 |  |  |  |  |  |  |  | 70 |  | 〇 | 〇 |  |  |  |  |  |
| 17 |  |  |  |  |  |  |  | 71 |  |  |  |  |  |  |  |  |
| 18 |  |  |  |  | 〇 |  |  | 72 |  |  | 〇 |  |  |  |  |  |
| 19 |  |  |  |  | 〇 | 〇 |  | 73 |  | 〇 | 〇 | 〇 | 〇 | 〇 |  |  |
| 20 |  |  |  |  |  |  |  | 74 |  |  |  |  |  |  |  |  |
| 21 |  |  |  | 〇 | 〇 |  |  | 75 |  |  |  |  | 〇 | 〇 |  |  |
| 22 |  | 〇 | 〇 |  |  |  |  | 76 |  |  |  |  | 〇 | 〇 |  |  |
| 23 | 〇 | 〇 |  |  |  |  | 〇 | 77 |  |  |  |  |  |  |  |  |
| 24 |  |  | 〇 |  |  |  |  | 78 |  |  |  |  |  |  |  |  |
| 25 | 〇 |  |  |  |  |  |  | 79 |  |  |  |  | 〇 | 〇 |  |  |
| 26 |  |  |  | 〇 | 〇 |  |  | 80 |  |  |  |  |  |  |  |  |
| 27 | 〇 | 〇 | 〇 |  |  |  |  | 81 |  | 〇 | 〇 |  | 〇 | 〇 |  |  |
| 28 |  |  | 〇 |  |  |  |  | 82 |  |  | 〇 |  | 〇 | 〇 |  |  |
| 29 |  |  |  | 〇 | 〇 |  |  | 83 |  |  |  |  | 〇 | 〇 |  |  |
| 30 | 〇 |  |  |  | 〇 | 〇 |  | 84 |  |  |  |  |  | 〇 |  |  |
| 31 | 〇 |  |  |  | 〇 |  |  | 85 |  |  |  | 〇 | 〇 |  |  |  |
| 32 |  |  |  |  |  |  |  | 86 |  |  |  | 〇 | 〇 |  |  |  |
| 33 |  |  |  |  |  |  |  | 87 |  | 〇 | 〇 |  |  |  |  |  |
| 34 |  |  |  | 〇 | 〇 |  |  | 88 | 〇 |  |  |  |  |  |  |  |
| 35 |  |  |  |  |  |  |  | 89 |  | 〇 | 〇 |  |  | 〇 |  |  |
| 36 |  |  |  |  |  | 〇 |  | 90 |  |  | 〇 |  |  | 〇 |  |  |
| 37 |  |  |  |  | 〇 |  |  | 91 |  | 〇 | 〇 |  |  | 〇 |  |  |
| 38 |  |  |  |  |  |  |  | 92 | 〇 | 〇 | 〇 | 〇 |  | 〇 |  |  |
| 39 |  | 〇 | 〇 |  |  |  |  | 93 | 〇 |  |  |  |  | 〇 |  |  |
| 40 |  |  |  | 〇 | 〇 | 〇 |  | 94 |  | 〇 | 〇 |  |  | 〇 |  |  |
| 41 |  |  |  |  |  |  |  | 95 |  |  | 〇 | 〇 | 〇 | 〇 |  |  |
| 42 |  |  |  |  |  |  |  | 96 |  |  |  |  |  | 〇 |  |  |
| 43 | 〇 |  |  |  | 〇 |  |  | 97 | 〇 |  | 〇 |  |  | 〇 |  |  |
| 44 |  |  |  | 〇 | 〇 |  |  | 98 |  |  | 〇 |  |  | 〇 |  |  |
| 45 |  |  |  |  |  |  |  | 99 |  |  |  |  |  | 〇 |  |  |
| 46 |  |  |  |  | 〇 | 〇 |  | 100 |  | 〇 | 〇 |  |  | 〇 |  |  |
| 47 |  |  |  | 〇 | 〇 |  |  | 101 | 〇 |  | 〇 |  |  | 〇 |  |  |
| 48 | 〇 |  | 〇 |  |  | 〇 |  | 102 |  |  |  |  |  | 〇 |  |  |
| 49 |  |  |  |  | 〇 | 〇 |  | 103 |  | 〇 | 〇 | 〇 | 〇 | 〇 |  |  |
| 50 |  |  |  |  | 〇 | 〇 |  | 104 | 〇 | 〇 | 〇 |  |  | 〇 |  |  |
| 51 |  |  |  | 〇 | 〇 |  |  | 105 |  | 〇 | 〇 |  |  | 〇 |  |  |
| 52 | 〇 |  |  |  |  |  |  | 106 |  |  |  | 〇 | 〇 | 〇 |  |  |
| 53 |  |  |  |  |  |  |  | 107 |  |  |  | 〇 | 〇 | 〇 |  |  |
| 54 |  |  |  |  |  |  |  | 108 |  |  |  |  |  | 〇 |  |  |

1) Risk of building collapse

2) Risk of flooding

3) Possibility of power loss

4) Instability of electricity supply

5) Instability of oxygen supply

6) Instability of water supply

7) Radiation protection system

Supplementary Table 2. Hospital support list

| **Medical institutions** | **Risk indicator** | | | | | | | **Medical institutions** | **Risk indicator** | | | | | | |  |  |
| --- | --- | --- | --- | --- | --- | --- | --- | --- | --- | --- | --- | --- | --- | --- | --- | --- | --- |
|  |  |  |  |  |  |  |  |  |  |  |  |  |  |  |  |  | |
|  | **i** | **ii** | **iii** | **iv** | **v** | **vi** | **vii** |  | **i** | **ii** | **iii** | **iv** | **v** | **vi** | **vii** | |  |
|  |  |  |  |  |  |  |  |  |  |  |  |  |  |  |  |  |  |
| 1 |  | 〇 |  |  | 〇 |  | 〇 | 55 |  |  |  |  |  |  |  | |  |
| 2 |  |  |  |  |  |  |  | 56 |  |  |  |  |  |  |  | |  |
| 3 | 〇 |  | 〇 | 〇 | 〇 |  |  | 57 |  |  |  |  | 〇 |  |  | |  |
| 4 |  |  |  |  | 〇 |  |  | 58 |  |  |  | 〇 | 〇 |  |  | |  |
| 5 | 〇 |  | 〇 |  |  |  |  | 59 |  |  |  |  |  |  |  | |  |
| 6 |  |  |  |  |  |  |  | 60 |  |  |  |  |  |  |  | |  |
| 7 | 〇 | 〇 | 〇 |  |  |  |  | 61 |  |  |  |  |  |  |  | |  |
| 8 |  |  |  |  | 〇 |  |  | 62 |  |  |  |  |  |  |  | |  |
| 9 |  | 〇 | 〇 |  |  |  |  | 63 |  |  |  |  |  |  |  | |  |
| 10 |  |  |  | 〇 |  |  |  | 64 |  |  | 〇 |  | 〇 |  |  | |  |
| 11 |  |  | 〇 |  |  |  |  | 65 |  |  |  |  | 〇 | 〇 |  | |  |
| 12 |  | 〇 | 〇 | 〇 | 〇 |  |  | 66 |  |  |  |  |  |  |  | |  |
| 13 |  |  |  |  |  |  |  | 67 |  |  |  |  |  |  |  | |  |
| 14 |  |  |  |  |  |  |  | 68 |  |  |  |  | 〇 |  |  | |  |
| 15 |  |  |  |  | 〇 |  |  | 69 |  |  |  |  |  |  |  | |  |
| 16 |  |  |  |  |  |  |  | 70 |  | 〇 | 〇 |  |  |  |  | |  |
| 17 |  |  | 〇 |  |  |  |  | 71 |  |  |  |  |  |  |  | |  |
| 18 |  |  |  |  | 〇 |  |  | 72 |  | 〇 | 〇 |  |  |  |  | |  |
| 19 |  |  |  |  | 〇 | 〇 |  | 73 |  | 〇 | 〇 | 〇 | 〇 | 〇 |  | |  |
| 20 |  | 〇 |  |  |  |  |  | 74 |  |  |  |  |  |  |  | |  |
| 21 |  |  |  |  | 〇 |  |  | 75 |  |  |  |  | 〇 |  |  | |  |
| 22 |  | 〇 | 〇 |  |  |  |  | 76 |  |  |  |  | 〇 |  |  | |  |
| 23 | 〇 | 〇 | 〇 |  |  |  | 〇 | 77 |  |  |  |  |  |  |  | |  |
| 24 |  |  | 〇 |  |  |  |  | 78 |  |  | 〇 |  |  |  |  | |  |
| 25 | 〇 |  | 〇 |  |  |  |  | 79 |  | 〇 |  |  | 〇 | 〇 |  | |  |
| 26 |  |  |  | 〇 | 〇 |  |  | 80 |  | 〇 |  |  |  |  |  | |  |
| 27 | 〇 | 〇 | 〇 | 〇 | 〇 |  |  | 81 |  | 〇 | 〇 |  | 〇 | 〇 |  | |  |
| 28 |  |  | 〇 |  |  |  |  | 82 |  | 〇 | 〇 |  |  | 〇 |  | |  |
| 29 |  |  | 〇 |  | 〇 |  |  | 83 |  |  |  |  | 〇 | 〇 |  | |  |
| 30 | 〇 |  |  |  | 〇 |  |  | 84 |  |  |  |  |  |  |  | |  |
| 31 | 〇 |  | 〇 |  | 〇 |  |  | 85 |  |  |  |  | 〇 |  |  | |  |
| 32 |  |  |  |  |  |  |  | 86 |  |  |  | 〇 | 〇 |  |  | |  |
| 33 |  |  |  |  |  |  |  | 87 |  |  |  |  | 〇 |  |  | |  |
| 34 |  |  |  | 〇 | 〇 |  |  | 88 |  | 〇 | 〇 |  |  |  |  | |  |
| 35 |  |  |  |  |  |  |  | 89 |  | 〇 |  |  |  | 〇 |  | |  |
| 36 |  | 〇 | 〇 | 〇 |  | 〇 |  | 90 |  | 〇 | 〇 |  |  | 〇 |  | |  |
| 37 |  |  |  |  | 〇 |  |  | 91 |  | 〇 | 〇 |  |  | 〇 |  | |  |
| 38 |  |  |  |  |  |  |  | 92 | 〇 | 〇 |  |  |  | 〇 |  | |  |
| 39 |  | 〇 | 〇 |  |  |  |  | 93 | 〇 | 〇 | 〇 | 〇 |  | 〇 |  | |  |
| 40 |  |  |  | 〇 | 〇 |  |  | 94 |  | 〇 | 〇 |  |  | 〇 |  | |  |
| 41 |  |  |  |  |  |  |  | 95 |  | 〇 | 〇 |  | 〇 | 〇 |  | |  |
| 42 |  |  |  |  |  |  |  | 96 |  | 〇 | 〇 | 〇 |  | 〇 |  | |  |
| 43 | 〇 |  |  |  | 〇 |  |  | 97 | 〇 |  |  |  |  |  |  | |  |
| 44 |  |  |  |  |  |  |  | 98 |  | 〇 | 〇 |  |  |  |  | |  |
| 45 |  |  |  |  |  |  |  | 99 |  | 〇 | 〇 | 〇 |  | 〇 |  | |  |
| 46 |  |  |  |  | 〇 |  |  | 100 |  |  |  |  |  |  |  | |  |
| 47 |  |  |  |  | 〇 |  |  | 101 |  |  |  |  |  |  |  | |  |
| 48 | 〇 | 〇 | 〇 |  |  | 〇 |  | 102 |  |  |  |  |  | 〇 |  | |  |
| 49 |  |  |  |  | 〇 |  |  | 103 |  | 〇 | 〇 | 〇 | 〇 |  |  | |  |
| 50 |  |  |  |  | 〇 |  |  | 104 | 〇 | 〇 | 〇 | 〇 |  | 〇 |  | |  |
| 51 |  |  |  |  | 〇 |  |  | 105 |  | 〇 | 〇 | 〇 |  | 〇 |  | |  |
| 52 | 〇 |  |  |  |  |  |  | 106 |  |  |  | 〇 | 〇 | 〇 |  | |  |
| 53 |  |  | 〇 |  |  |  |  | 107 |  | 〇 |  |  | 〇 |  |  | |  |
| 54 |  |  |  |  |  |  |  | 108 |  |  |  | 〇 |  |  |  | |  |

1) Risk of building collapse

2) Risk of flooding

3) Possibility of power loss

4) Instability of electricity supply

5) Instability of oxygen supply

6) Instability of water supply

7) Radiation protection system
